# Complexification of In Vitro Models of Intestinal Barriers, A True Challenge for a More Accurate Alternative Approach

**DOI:** 10.3390/ijms24043595

**Published:** 2023-02-10

**Authors:** Michelle J. Haddad, Wendy Sztupecki, Carine Delayre-Orthez, Larbi Rhazi, Nicolas Barbezier, Flore Depeint, Pauline M. Anton

**Affiliations:** 1 Transformations et Agroressources, ULR 7519, Institut Polytechnique UniLaSalle, Université d’Artois, 60000 Beauvais, France; 2 HCS Pharma, 59120 Loos, France

**Keywords:** intestinal barrier, 3D co-cultures, innate and immune response, enteroendocrine cells, goblet cells, enterocytes

## Abstract

The use of cell models is common to mimic cellular and molecular events in interaction with their environment. In the case of the gut, the existing models are of particular interest to evaluate food, toxicants, or drug effects on the mucosa. To have the most accurate model, cell diversity and the complexity of the interactions must be considered. Existing models range from single-cell cultures of absorptive cells to more complex combinations of two or more cell types. This work describes the existing solutions and the challenges that remain to be solved.

## 1. Introduction

Among the three main points of entry of exogenous substances into the human body, along with the skin and the respiratory system, the gastrointestinal tract represents a significant playfield through oral exposure and is one of the most studied so far due to its ability to absorb nutrients and face noxious stimuli on an everyday basis. The main organ facing exchanges with the external environment is the small intestine, and it is the main site for the absorption of nutrients, oral drugs and chemical or biological pathogenic molecules. Characterization and evaluation of physiological modifications of the organism have long been studied in whole living organisms ranging from invertebrates to higher animal species and humans. For many years now, and mainly for ethical reasons, or to avoid the difficulties of extrapolating results between laboratory animals and humans, scientists have been developing alternative methods to limit the use of living animals, especially for the screening of new therapeutic molecules or to understand the toxic effects of environmental pollutants and their mechanisms of action. Alternative methods include in vitro and in silico approaches. In vitro models range from cell cultures to organoids with various levels of complexity depending on the question addressed, although using a cell culture is an essential step to evaluate the impact of a molecule. The main advantage of cell cultures is that they generally are easy to set up and allow researchers to obtain many data in a reasonable period of time. Cell cultures have been developed to investigate all functions of the body. However, unlike 3D organoid cultures that resemble more physiological tissue organization and functionality, most cell culture models are quite simple. They are generally conducted with human or animal cell lines, often immortalized to be able to multiply, but also stabilized to react homogenously after several passages.

The method of investigation of gut barrier functionality is largely dependent on the scale or level of interest, from cellular to tissue or full organism. It can range from the measurement of a permeability marker in the blood circulation of living animals or in humans to the study of cell lines or primary cultures of different origins, but also includes the use of ex vivo systems such as Ussing chambers requiring intestinal tissues, biopsies or surgical specimens.

In this review, we will present the literature on the state of the art of cellular models developed to investigate static in vitro intestinal barrier functionality and the future challenges to improve their accuracy and representativity of physiological events. The use of dynamic models, or of the static models for evaluating transport and metabolism, will not be detailed. The main goal is to have alternative and reproducible in vitro cellular models representing with accuracy the physiological interactions of this complex organ.

## 2. Organization and Functionality of the Intestinal Barrier

### 2.1. Role of the Intestinal Barrier

The intestinal barrier is known as the protective component of the gut shielding individuals against microbial or toxic insult. According to Bischoff et al. [1], the intestinal barrier is a functional entity separating the external environment (gut lumen) from the inner host, and consisting of mechanical elements (mucus, epithelial layer), humoral elements (defensins, immunoglobulins A), cellular immunological elements (lymphocytes, innate immune cells), muscular and neurological elements. The importance of the intestinal barrier mainly relates to the surface of the body concerned. Indeed, the small intestine, is a layer around 20 µm thick with a large surface area thanks to the villi and crypt. The colonic layer, by contrast is 20 times thicker [2]. Overall, the surface area of the human digestive tract is around 32 m^2^, corresponding to half of a badminton court according to Helander and Fändrik [3]. The intestinal barrier thus has two critical functions: (1) filtering, allowing only needed nutrients and other beneficial substances to pass; and (2) protecting, preventing harmful entities from reaching the blood circulation and attacking the body.

Whenever authors investigate the intestinal barrier, the impacts on health of substances entering the gut are often related to evaluation of alterations of intestinal permeability, whether the substances are nutrients, drugs or toxic substances. Over the past ten years, there have been more than 6500 articles about such evaluations reported in PubMed. Most of the time, these investigations have been restricted to evaluation of alterations specifically of epithelial permeability, but not overall intestinal permeability. However, this approach is quite limiting since it does not reflect the complexity of all the interactions happening physiologically. Indeed, as described above, the mucosa contains other key elements interconnected to each other and in charge of realizing these complex and almost perfect filtration and protection functions. It has been largely acknowledged by the scientific community that alteration of the gut microbiota composition, of the mucus layer structure or the presence of epithelial damage, are responsible for alteration of intestinal permeability. This leads to the translocation of the intestinal content to the inner layers of the intestinal wall and their subsequent absorption in the blood circulation, jeopardizing health. The alteration of intestinal permeability is also associated with the activation of the innate and adaptative immune responses of the gut.

### 2.2. Organization of the Intestinal Barrier

**Figure 1:**
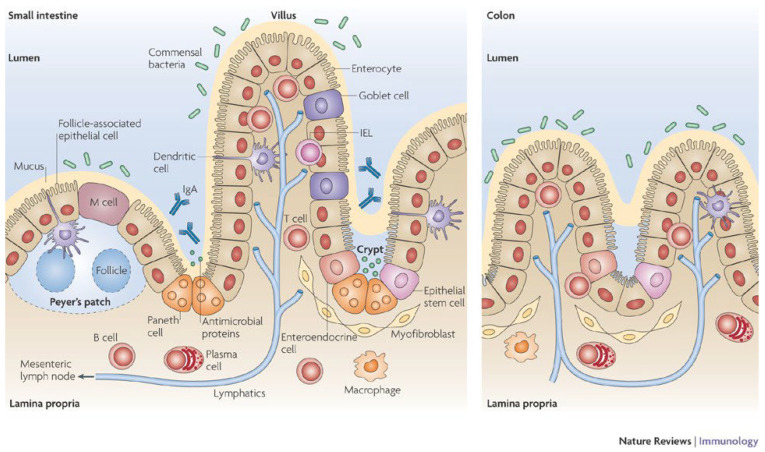
Schematic representation of intestinal epithelium cells and their localization [4].

The interactions between the microbiota, the epithelial and immune cells are then of great importance when studying the intestinal barrier function (Figure 1). At the organism level, their organization is as follows.

The gut microbiota: In humans, this consists of over 100 trillion bacteria, fungi, archaea and viruses representing globally a genome 100 times more important than the human genome. The microbiota composition (density and diversity) is important to keep the intestine clear from pathogens, to educate the immune system response and to help to metabolize correctly the nutrients that get into the lumen [5]. The microbiota settles at birth and will densify and diversify during the first three years, at which point its composition reaches that of adulthood [6]. However, the intestinal ecosystem remains quite malleable since many factors, among which are diet composition [7], stress [8], sleep deprivation and circadian disruption [9,10], may modify the overall microbiota profile.

The intestinal epithelium: Covering the villi, this epithelium is made of a single-cell lining rich in enterocytes or colonocytes, interspersed by goblet cells, as well as enteroendocrine cells (spread throughout the gut epithelium, representing 1% of the epithelial cells), M cells and occasionally tuft cells (chemosensory cells whose number is increased at times of parasitic infections). These epithelial cells are connected to each other thanks to three components from the basal to the apical membrane: desmosomes, adherens junctions and tight junctions [11]. While adherens junctions and desmosomes contribute to the stability of the epithelium, tight junctions are dynamic multiprotein complexes in charge of regulating the flow of water, ions and small molecules through the selective/semipermeable paracellular barrier. The intestinal epithelium mediates selective permeability via two major routes: (a) the transepithelial/transcellular, and (b) the paracellular pathways depending on the type and the size of molecules present in the lumen. In the crypts, we find stem cells in charge of renewing the epithelial lining, as well as, in the small intestine, Paneth cells secreting anti-microbial substances. These cells release, into the lumen, digestive enzymes, growth factors and antimicrobial peptides (AMP), including cryptdins (in mice) α-defensins (HD-5 and HD-6) and lysozyme, phospholipase A2 and proteins [12]. They can sense enteric bacteria via cell toll-like receptor (TLR) activation, triggering antimicrobial factor expression, and they discharge microbicidal granule content in the gut lumen. TRL5 is specific to Paneth cells, they express also low levels of TLR2 and undetectable levels of TLR4, 7 and 9 [13]. They are long-living cells with a slow turnover rate, differentiated from the stem cells situated in the bottom of the crypts, and are important for maintaining the functionality of stem cells which regenerate intestinal epithelium. The colonic crypts are far less complex, containing only stem and goblet cells on top of colonocytes. Enterocytes oversee the absorption of nutrients, such as simple carbohydrates, peptides and amino acids, and lipids, but also ions and water, thanks to transporters located at the brush border. The small intestine brush border is studded with long, densely packed microvilli [14]. They also release antimicrobial proteins such as β-defensins and cathelicidins in charge of disrupting pathogenic microorganisms’ membranes. They express brush border enzymes such as maltase, sucrase-isomaltase, lactase and peptidases, which are integral membrane proteins. Tight junctions between enterocytes lead to a physical barrier against microbial penetration. Colonocytes are similar, but their role is slightly different. They are in charge of the absorption of water, electrolyte, and various bacterial metabolites from the lumen to the bloodstream [15]. However, unlike enterocytes, these cells do not carry brush border enzymes and have short, irregular microvilli [14]. They use butyrate as a main source of energy for metabolic activity, thus expressing the monocarboxylate transporter 1 (MCT1) and a selective store-operated calcium channel, the membrane spanning 4-domain A12 (MS4A12) [16]. Next to them sit the goblet cells secreting antimicrobial peptides but also mucus, a heavily glycosylated and polymerized gel made of mucins. The secretion is different between the small intestine, protected by a single liquid mucus layer, and the colon, which has two, a liquid layer and a more viscous one [17]. Enteroendocrine cells (EEC) are able to release many hormones and other components in charge of controlling food behavior through gut–brain interactions, such as Glucagon-like peptide 1 (GLP-1), peptide YY (PYY), Cholecystokinin (CCK) or Serotonin (5-HT) [18]. Finally, M cells also sit at this level, generally overlying lymphoid follicles. They are in charge of capturing and transferring to dendritic cells (DC) luminal particles and antigens, thus inducing either mucosal immune responses or mucosal tolerance (for review see [19,20]).

The intestinal immune system is in charge of the induction of the innate and adaptative immune responses through an inflammatory response in order to prevent microbial penetration across the epithelium. The first line of defense belongs to the innate immune system, including macrophages and granulocytes allowing phagocytosis and the release of inflammatory mediators. Another type of intestinal innate immune cells are mast cells. They can release, once activated, a powerful array of proinflammatory mediators, among which are cytokines, histamine or serotonin. To complete this immunological innate protection, the intestine is also equipped with another group of immune cells in charge of the adaptative immune response. Antigen-presenting cells (APC), such as dendritic cells and macrophages, upon stimulation, orchestrate the interactions between the innate and adaptative immune systems. Dendritic cells are found throughout the intestine, including the lamina propria, the isolated lymphoid follicles, the Peyer’s patches and the mesenteric lymph nodes [21,22]. This adaptative response relates to the release in the lumen of IgA by plasmocytes, but also of an appropriate maturation of naive Th lymphocytes into Th1, Th17, Th2 and iTreg cells upon specific cytokine stimulation. Finally, the gamma/delta intestinal epithelial lymphocytes, closely associated with the basolateral membrane of the intestinal epithelial cells, are involved in the maintenance of the intestinal barrier [23].

### 2.3. Consequences of the Disruption of the Intestinal Barrier

Alteration of the settlement of the intestinal barrier in infancy, or its disruption later in life, will have various consequences. Disruption of the epithelial barrier following bacterial infection or tissue damage induces the release of anti-microbial peptides abrogating the tolerogenic properties of dendritic cells and the activation of macrophages. An immune intestinal dysfunction is critical in the predisposition to, and exacerbation of, numerous immune and inflammatory conditions ranging from the acute, such as gastroenteritis, to chronic responses, including inflammatory bowel disease (IBD), food allergies, coeliac disease and diabetes [24]. The crosstalk between the gut microbiota, the epithelial layer, the gut immune system, the enteric nervous system and the brain will also contribute to the modulation of the intestinal homeostasis and intestinal permeability [8,25]. However, due to the very complex and inter-organ interactions that take place, this will not be studied in this review.

The following sections will focus on the descriptions and limits of the current models of single-cell culture or co-culture and the remaining challenges to better simulate physiological (absorption, metabolization, etc.) as well as pathological conditions (permeability alteration, etc.) and to more closely mimic the immune intestinal dysfunctions observed during auto-immune and inflammatory conditions.

## 3. Cellular Models Used to Test the Functionality of the Intestinal Barrier

When developing cell culture models, researchers often use immortalized cancer cell lines that can be cultivated for many generations without major perturbation of the phenotype. However, they may not express typical functions of the original tissue. In contrast, most primary cells require technical skills to harvest and have a very limited lifespan corresponding to the original cell type. As an alternative, stem cells can be cultivated for about 40 generations before they reach senescence, and fetal cells have wider proliferating potential, being multipotent cells. Another option is to immortalize normal cells by transfection with temperature-sensitive simian viral protein (SV40) large T antigen. Cells then retain normal phenotype while their proliferation potential is regulated by the temperature-sensitive plasmid. In the remaining parts of the review, we will be covering the different options that can be found in the literature.

### 3.1. Single-Cell Models Mimicking the Intestinal Physical Barrier

In this part, we will describe the different cell models of the intestinal barrier currently used for food, toxicant or drug studies. We will focus first on human cell lines, but other mammal cell lines will be discussed also because they can be useful models for human digestibility studies.

#### 3.1.1. Absorptive Cells: Enterocytes and Colonocytes

The gold standard for studying in vitro the changes in the epithelium due to exposure to nutrients, functional ingredients, toxicants or drugs is to work with absorptive cell lines expressing an enterocyte or colonocyte phenotype (Table 1) because they represent more than 80% of the intestinal epithelial cells [26].

##### Enterocyte Cell Lines Coming from Human Cancer Cells

The Caco-2 cell line, derived from human colorectal adenocarcinoma [75], can differentiate spontaneously into polarized enterocytes with brush border functions and express most of the typical enzymes naturally present at the surface of enterocytes 14 to 21 days after reaching confluence [32]. They are used for a wide range of applications, including studies of intestinal transport, absorption, bioavailability and permeability of intestinal barrier to food and drug components. Since the first passage, there has been some diversification of the phenotype [33]. One specific clone, the Caco-2-TC7, resulted from a late passage (198) of the Caco-2 parental cell line [37]. Its main advantage is a more homogeneous phenotype and thus a more stable response to stimuli. It grows under the same culture conditions and differentiates faster than the parental line. In addition, it presents a more developed intercellular junction when fully differentiated, which is why it is more and more used for intestinal permeability studies. However, as it exhibits higher expression of brush-border-associated enzymes than the human jejunum cells, the model may overestimate absorptive capacity. Other clones of Caco-2 have been developed over the years for very specific scientific questions, but their characteristics are not described as thoroughly in the literature (Table 1). Another cell line derived from a human colon carcinoma primary tumor, the HT29, is often used to model enterocyte functionality [38]. The HT29 cells are interesting as they can, depending on the stimulus, differentiate into and express different phenotypes. Cultured in RPMI-1640 medium and high glucose, they will stay undifferentiated. If transferred to glucose-free DMEM supplemented with galactose, they start to polarize and acquire enterocyte-like phenotype, although this differentiation is reversible. Permanent tight junctions and barrier functions can be acquired after differentiation with sodium butyrate, while a goblet-like phenotype and Mucin 2 (MUC2) secretion is the consequence of methotrexate stimulation, as described in the next section. The HT29 cell line is used in studies of cell differentiation, food digestion and bioavailability, and cell mechanisms. They can also be used for studies of drug and food transport, intestinal immune response to bacterial infection and microorganism survival, adhesion or invasion [39]. As enterocyte-like tissue, they synthetize brush border enzymes, including hydrolases, and they secrete metabolites, cytokines and growth factors. However, their enzyme expression is lower than normal intestinal cells and they do not express lactase and maltase-glucoamylase. These differences in enzyme expression, together with the very slow differentiation rate (30 days for HT29, 15–21 days for Caco-2), affect the suitability of the model in reflecting in vivo permeability. Indeed, the Caco-2 cell line shows TEER values four times higher than HT-29. As part of the stabilized cell lines derived from HT-29, we can cite WiDr, used for tumorigenesis studies as xenografts on nude mice; HT29-MTX and HT29-18N, carrying a goblet cell phenotype when cultivated with methotrexate or carbachol, respectively; HT29-FU, being chemoresistant and generally used for carcinogenesis studies; and HT-29 18C, which may lack sucrase-isomaltase when cultivated in a medium containing glucose [39,43,76].

##### Enterocyte Cell Lines Coming from Human Normal Cells

The analysis of the functionality of enterocytes based on cancer-derived cell lines may have some drawbacks linked to the activation of intracellular pathways characteristic of tumorigenic cells. That is why some models use human fetal intestinal cells. HIEC-6 are normal human small intestinal crypt cells initially isolated from fetal small intestine [46]. They are used to study the interactions between pathogens and the intestinal epithelium, and more specifically its cell self-renewal and differentiation. They keep their characteristics for 30 passages and they can be converted into robust crypt base columnar (CBC)-like cells with a combination of Wingless-related integration site (Wnt-3a), R-spondin 1 and noggin, which increase their proliferation rate [47]. H4 cells are a non-transformed primary human fetal epithelial line. It is a useful model to study fatty acid transport and absorption [52] but also inflammation response, transportation of immunoglobulin in amniotic fluids and the pathophysiology of necrotizing enterocolitis (NEC) [53]. However, this immature human small intestine can respond excessively to external inflammatory stimuli compared to small intestine from infants and children [54], and they do not form any tight junctions, nor do they polarize [48]. FHs-74-Int, also cited as tsFHI, is derived from normal human fetal intestine and transfected with SV40. Since these cells have been reported to show mature epithelial-like characteristics, this cell line is considered to be a suitable model for proliferation, immunomodulation and permeability studies [49]. Due to temperature-sensitive sv40 regulation, the cells can proliferate at 32 °C and, when transferred to 37 °C, the sv40 antigen breaks down and cells differentiate into an immature enterocyte-like monolayer but do not polarize or form tight junctions [48]. The use of fetal cell lines may be controversial for ethical reasons, but, because of their pluripotency, these cells have high expansion rates, phenotypic plasticity, they are more tolerant to cryopreservation and they can retain phenotypic characteristics longer than other normal cell lines [50]. Other cell lines may also be used, as described in Table 1. Whether working with primary cultures, stem cells or fetal cells, the protocols require very specific growth conditions that make them mostly unsuitable for co-culture.

##### Colonocyte Cell Lines Coming from Human Cancer Cells

T84 cells are malignant human colonocytes [77] that can differentiate into crypt-like cells after induction with TGFβ1. Furthermore, in a study using this cell line, the authors observed no release of proinflammatory markers (IL-8 and TNF-α) after LPS induction [59]. This hypo-responsiveness to stimulation can be an advantage to model a noninflamed intestinal mucosa. They also hold interest for studying hormonal control of human carcinoma cell growth, mechanisms of electrolytes transport, intestinal permeability, signaling pathways related to inflammatory response and bacteria/colonocyte interactions [39]. SK-CO15 are colorectal cell lines [78,79]. Their main interest specifically relates to the study of ion exchange at the apical level [57]. DLD1 cells are another human colon carcinoma that can be reprogrammed to obtain pluripotent stem cells and create 2D and 3D models for therapeutic studies for cancer [80]. SW620, Colo-205, HCT116 and HRT118 also are colorectal cancer cell lines. These cell lines are currently used in cancer research and toxicology [60,62,64].

##### Colonocyte Cell Lines Coming from Human Normal Cells

The cells of the NCM460 cell line are sv40-immortalized human transverse colonic cells [73]. They are very close to the normal colonocytes and can be used for in vitro studies of the intestinal barrier for non-transformed colonocytes, but also to study absorption. CCD18-co (CRL-1459) can also be used as human non-cancerous colonocytes. The cells have a fibroblast morphology [74]. Finally, FHC (CRL-1831), which is a fetal cell line with the epithelial morphology of normal colonocytes [74], may also be reprogrammed to obtain pluripotent stem cells to create 2D and 3D models for therapeutic studies for cancer [80].

Here, we reviewed some human monoculture models used to study the consequences of nutrients, toxicants or drugs in terms of their absorption or interaction with the intestinal epithelial barrier. Although we restricted this review of the literature to human cell lines, there also exist murine and porcine cell lines [81,82,83,84]. The principal disadvantage of monoculture models is that they do not reflect the diversity of the mucosa with its several cell types and multiple functions. This drawback includes a critical physical element of the mucosa, that the mucus layer is acting as a physical and chemical defense against pathogens and molecules. As a physical barrier, it protects cells from pathogenic bacteria. Furthermore, it also has protective and immune-regulatory properties thanks to the presence of antimicrobial peptides and cytokines secreted by the epithelial cells. To better study absorption through intestinal epithelium, bioactive molecule solubility in the mucus layer should also be considered.

#### 3.1.2. Secretory Cells: Goblet, Paneth, Enteroendocrine and Tuft Cells

As briefly mentioned above, secretory cells present at the surface of the small and large intestine interact with the enterocytes and the colonocytes to improve the physical and biochemical barrier function with complementary activities. The need for cellular models mimicking these specificities is of great importance to understand the role of the mucus layer in the maintenance of the intestinal barrier as well as the links between mucus layer dysfunctions and related diseases, or the regulation of intestinal hormonal secretions by bacterial metabolites, for instance (Table 2).

##### Mucin-Secreting Goblet Cells

Goblet cells normally represent 10% of the intestinal epithelium, and they produce a mucus of variable viscosity depending on the gut segment and made of highly glycosylated proteins called mucins (MUC). Mucus also harbors adherent bacterial species. Goblet cells are easily identified thanks to secretory granules present in their cytoplasm. Among the many mucins secreted, MUC2 is the one most represented, but in the presence of parasitic or bacterial infections there also are secretions of MUC3 and MUC5AC [101]. Physiologically, goblet cells secrete mucins to ease the bowel movements and protect the gut from irritating components of the food matrix, but also in response to irritating gases, nerve activation, reactive oxygen species (ROS) and inflammatory mediators, or to changes in the microbiota composition sometimes linked to bacterial infections. Excessive mucin secretion can be linked with inflammation caused by bacterial infection [102]. While most of these events may not be easily reproducible with static approaches, their role in the characterization of intestinal barrier function cannot be neglected. The most frequently used cellular model of goblet cells is the HT29-MTX cell line. These cells are a sub-strain of the colorectal carcinoma-derived cell line HT29 [87] treated with a high concentration of methotrexate [88]. Because of their mucus-secreting phenotype and their adhesive properties, they have been used to study the transport and properties of bioactive compounds, as well as microorganisms’ adhesion and survival. HT29-MTX cells produce MUC2 secretion and, to a greater extent, MUC5AC secretion, which renders them an appropriate model to study the role of mucus in the intestinal barrier function. Generally, they are not used alone but rather in co-cultures, as explained below, but may under specific circumstances be used to study the effect of mucus on the permeation of drugs [89]. The LS174T is another human intestinal epithelial cell line that comes from cells isolated from the colon of a patient suffering from a stage 2 colorectal cancer. This is a trypsinized variant from the LS180 cell line which also secretes mucus. LS174T cells produce higher levels of carcinoembryonic agents than its parental LS-180 cell line and presents faster doubling time but secretes lower amounts of mucin [90]. They are used when investigating mucus activity as a physical barrier and more specifically mucin secretion regulation [102,103,104,105]. Furthermore, in contrast to the HT29-MTX cell line, the LS174T cells express high levels of MUC2 mRNA and lower levels of MUC5AC mRNA [104]. Finally, LS174T cells grow unorganized and inadherent [42], which is their main limitation as compared to the HT29-MTX cell line. However, from the literature it seems that adhesion and high levels of mucus secretion may be difficult to have appear together since it has been observed that adherent cell lines secrete less mucin than the others [106].

##### Paneth Cells

While dysfunction of Paneth cells, typically of the small intestine, can contribute to the pathogenesis of chronic inflammatory bowel disease [107], they are not currently able to be cultured without other epithelial or stem cells according to the review of Lueschow et al. [12]. One paper, however, described the autonomous growth of single Lgr5 stem cells into crypt-like structures with de novo generated stem cells and Paneth cells at their bottom in a matrigel-based culture system containing EGF, the Wnt agonist R-spondin1 and the BMP inhibitor Noggin4 [108]. At present, for these technical reasons, most studies of Paneth cells are usually run from murine intestinal crypt cells [109].

##### Enteroendocrine Cells

Although enteroendocrine cells (EEC) represent less than 1% of the overall epithelium cell population, they are the starting framework to measure the gut hormonal responses to food intake. Physiologically, there are three prevalent main enteroendocrine cell types in the lower gastro-intestinal tract, namely, EC cells, D cells and L cells [110], which have different endocrine capacities. Due to these specificities, in vitro models are scarce and difficult to set up mainly because of their distribution throughout the gut and their low proportion as compared to the other cell types. So far, to our knowledge, there are few cell lines of human origin. The NCI-H716, an enteroendocrine L cell line, comes from cecal adenocarcinoma [92], and the HuTu-80 was isolated from duodenal adenocarcinoma and also expresses an L enteroendocrine phenotype. Once differentiated, the NCI-H716 cells respond homogenously and express GLP1, PYY and CCK [93,94]. To attach to the culture plate, they need a basement membrane coating [93]. By contrast, little information is given for HuTu-80 cells apart from the fact that they release PYY to a lesser extent than NCI-H716 cells [95]. Furthermore, HuTu-80 sensitivity to short chain fatty acids (SCFAs) seems to be lower than that of the other line [95]. Two other enteroendocrine cell lines are of murine origin. The Secretin Tumor Cell, STC-1, line comes from duodenal tumor cells of a transgenic-mouse that grow well in DMEM and, when properly cultivated, express a phenotype of distal intestinal L-cells. It is routinely used to study GLP-1 secretion. Because they are able to secrete numerous gut hormones, such as cholecystokinin (CCK), glucose-dependent insulinotropic polypeptide (GIP), PYY, pancreatic polypeptide (PP), neurotensin (NT), GLP-1, GLP-2 and oxyntomodulin (OXM), they remain a popular choice for primary screening of substances having the potential to modulate the enteroendocrine cell response. In addition, nutrients added to the media can modulate STC-1 gene expression, making STC-1 proglucagon fragment processing in an intermediate manner between intestinal L-cells and pancreatic α-cells [96]. The other murine cell line expressing the enteroendocrine phenotype is GLUTtag [100]. Compared to the previous cell line, this one differentiates partially and expresses pro-glucagon over a 4–8 week period as well as GLP-1 and CCK, but not PYY. Thanks to that, and despite the fact that they are of rodent origin, they remain widely used since they respond to the same stimulus that triggers GLP-1 secretion in humans.

When cultivablein vitro, all these cell types may adapt to media in which enterocyte or colonocyte cells may grow, although conditions may need to be adapted. The following section will detail the conditions under which cells of different phenotypes may be grown together.

### 3.2. Advanced Models to Investigate Gut Integrity

The use of co-culture models has improved the in vitro study of the organization and functionality of the gut barrier under physiological as well as pathological conditions. These models associate different combinations of the abovementioned intestinal cell lines and frequently use insert systems. Cultures of epithelial cells on inserts allows for their polarization and free access to both the lumen/apical (represented by the inside of the insert) and *lamina propria*/basal compartments. Furthermore, the inserts may be coated with matrigel to allow the adherence of some cell types, such as immune cells, to the membrane. By contrast, 3D-organoid cultures from isolated crypts are not grown on inserts but in domes of matrigel placed at the bottom of the well.

These improved co-culture models using mainly cells of human origin are attracting high levels of interest because, since they mimic the physiology more closely, this allows a reduction in animal experimentation. This is well perceived for ethical reasons but also for scientific reasons because of the discrepancies in responses between species and the difficulty of extrapolating results to humans [111]. The ultimate goal will be to obtain an intestinal barrier close to the intestinal mucosa containing about 85% epithelial cells, 10% mucus-secreting cells, 1% enteroendocrine and tuft cells and variable numbers of immune-related cells.

#### 3.2.1. Investigation of the Epithelial Protection by the Mucus Layer

The main cell types involved in the maintenance of the integrity of the intestinal barrier are epithelial cells and mucus-secreting goblet cells. As already mentioned in the first paragraph, these two types of cells coexist and interact in the digestive system. Examples of systems reproducing these interactions by co-cultivation using different cell types will be discussed in this section. In order to test drug, toxicant and nutrient transport, metabolism and bioavailability across the intestinal epithelium, the presence of both absorptive and goblet cells is required to mimic the physiological features of the intestine.

Caco-2/HT29-MTX or Caco-2-TC7/HT29-MTX: The traditional co-culture model to investigate this epithelium protection is the combined cultivation of fully differentiated enterocyte-like Caco-2 cells, or their clone Caco-2-TC7, with mucus-secreting cell lines such as HT29-MTX or LS174T. Regarding the Caco-2/HT29-MTX co-culture, both these two cell types have been described as having different requirements for optimal growth and function, among which is an appropriate seeding ratio [112]. Different ratios have been evaluated: 75/25 [113]; 90/10, 80/20, 70/30 [114]; and 75/25, 50/50, 25/75 [115]. The use of the 90/10, 80/20 and 75/25 ratios reflects the physiological evolution of goblet cell numbers throughout the gut changing from 10% in the duodenum [112] to 24% in the distal colon [116]. The 90/10 ratio is also the one most widely used to mimic the human intestinal barrier in both absorption and permeability studies [112]. Indeed, Caco-2 cells are often used to study toxin-induced effects on human epithelial barrier functions [117], and HT29-MTX cells are mainly used to study bacterial invasion and adhesion [118]. This co-culture Caco-2/HT29-MTX is also considered to be an appropriate model for studying drug absorption and for permeability studies [119,120,121]. This is mainly due to the fact that the presence of the mucus layer coming from the HT29-MTX cells acts as an important barrier against the passage of pathogens and toxins across the intestinal membrane [87,122], although some bacterial strains such as Salmonella may use the mucus to enhance their pathogenicity [118].

Caco-2/LS174T: The other mucus-secreting cell line that may be used with enterocyte-like cells is the LS174T [123]. Along with Caco-2 cells, this model is used to investigate cell-type-specific mucin expression mechanisms [124], as well as mucin expression in colon cancer [104]. Such a co-culture is aimed at evidencing the protection of the intestinal epithelial barrier [125]. However, with such an in vitro approach, the fine characterization of the mucus secretion may be questioned since the Caco-2 and LS174T cell lines may both be expressing MUC5AC mRNA, with Caco-2 exhibiting the highest level. By contrast, MUC2 mRNA was strongly expressed in LS174T while the authors hardly detected it in Caco-2 [104]. Furthermore, another study confirmed MUC2 secretion by LS174T but not Caco-2 [124]. Both these models of co-culture may be set up in 3D systems such as Transwell^®^ inserts.

To our knowledge, there is no specific model to investigate epithelial protection by the mucus layer at the colonic level due to the absence of compatible cell lines (no publication using the T84/HT29-MTX co-culture). To overcome this, the only solution proposed so far is to modify the Caco-2/HT29-MTX ratio to increase the mucus layer thickness [112,126].

#### 3.2.2. Investigation of the Modulation of the Innate Intestinal Immune Response of the Intestinal Barrier

Over the past 20 years, interest in improving the intestinal barrier models by including immune cells in cultures has been growing [127]. The main innate immune cell lines used to simulate the interaction with epithelial cells are macrophage cell lines of human (THP-1) or murine (RAW 264.7) origins (Table 3). THP1 are pro-monocytic cells which may become adherent to culture plates and transform into macrophages in the presence of phorbol-12-muristate-13-acetate (PMA). They can also differentiate into dendritic cells with serum-free medium supplemented with IL-4, Granulocyte macrophage colony-stimulating factor (GM-CSF), TNF-α and inomycin [128]. Their genetic background is homogenous, and this cell line represents a simplified model to study monocyte–macrophage polarization, and they have a growing rate higher than Peripheral Blood Mononuclear Cells (PBMC). RAW 264.7 cells are widely used to study oxidative stress and inflammatory and antibacterial activities because they mimic murine bone-marrow-derived macrophage properties with similar surface receptors, the toll-like receptors (TLR). However, as with many of the cell lines, the phenotype may change with consecutive passages [129,130].

Caco-2/THP1: Over the last 15 years, several in vitro models were constructed to analyze the interaction between intestinal epithelial and macrophage-like cells. The choice to use THP-1 cells in these models was prevalent because most macrophages found in the healthy intestine are characterized by the absence of several response receptors, such as the LPS-receptor CD14 [135], as well as the absence of an inflammatory response towards pathogens [136]. Furthermore, these cells can be grown in vitro up to passage 25 with no eventual changes in cell sensitivity [128]. In this context, Caco-2 cells may be co-cultivated with THP-1 cells to evaluate the chemical interactions existing between these two cell types during gastrointestinal inflammation [120,137]. The Caco-2 cells are seeded on the apical side of the Transwell^®^ inserts and the immune cells are grown in the basolateral compartment, as with most of the co-culture models nowadays [137], though there are some exceptions where the intestinal and the immune cells are seeded in the same compartment [138]. This model closely mimics the physiological conditions and provides a more compatible setup to evaluate drug permeability in vitro [139]. However, the cells’ seeding densities differ among laboratories, which leads to variable outcomes. These variable responses are responsible for the difficulties associated with establishing a co-culture model combining immune cells with epithelial ones [135].

Caco-2/RAW 264.7: Other studies have associated human intestinal epithelial-like Caco-2 with murine RAW 264.7 macrophages to look into the anti-inflammatory properties of different types of molecules. Thanks to such a model, authors were able to identify the molecular mechanisms down-regulating the expression of pro-inflammatory mediators excessively produced by RAW 264.7 macrophages under stimulated conditions [140].

Caco-2/HT29-MTX/THP-1 or Caco-2/HT29-MTX/RAW 264.7: In all the tri-culture setups that have been described, the culture system was established by growing Caco-2 and HT29-MTX cells onto inserts with semipermeable membranes, and RAW 264.7 or THP-1 cells in the basolateral compartment where the macrophage stimulus was added. Both these two macrophage cell types require the same culture medium, RPMI 1640 [129,141], although THP-1 cells grow in suspension and RAW 264.7 differentiate as an adherent cell line [142]. However, Raw 264.7 should not be used after passage number 30, otherwise it might have an impact on their stability [142]. Most of the studies conducted with this system have concluded that this tri-culture model can mimic the intestinal barrier response during gut inflammation in vivo as a decrease in the TEER is observed. This approach may provide further insight into the integrity of the intestinal membrane [143].

Finally, neutrophil activity may be investigated thanks to the use of the **HL-60** promyelocytic leukemia cell line able to differentiate into neutrophilic promyelocyte in an appropriate RPMI-1640 medium containing FBS, L-glutamine and HEPES (Table 3). HL-60 cells are also used as a macrophage-like cell model to study the anti-inflammatory potential of bioactive compounds [134].

#### 3.2.3. Investigation of the Modulation of the Adaptative Intestinal Immune Response of the Intestinal Barrier

Other co-culture models have been developed to investigate the adaptative immune response in the presence of nutrients, toxicants or drugs in the intestinal lumen. **Raji** cells express a lymphocyte phenotype and come from human Burkitt’s lymphoma which presents Fc receptors for binding immunoglobins (Table 3). In co-culture with Caco-2, they can induce enterocyte transformation into M-cells [144,145,146].

Caco-2/Raji and Caco-2/HT29-MTX/Raji: Some studies have been undertaken with Caco-2 cells in association with the Raji cells [147] to mimic the events happening in M cells in the Peyer’s patches. This approach was followed by different laboratories to study the transport and uptake of luminal antigen mechanisms by M cells via an improved in vitro model of human-follicle-associated epithelium (FAE) [148]. M cells are normally in charge of trapping the antigen to present it to APCs, the macrophages, dendritic cells and B-lymphocytes all situated in the Peyer’s patch with T-lymphocytes. M cell models are often used for mucosal vaccine development and to study their interactions with bacteria. There are no M cell tissue cell lines available and primary M cells are not easily purified from tissue. That is why a differentiation of enterocytes is necessary to create M-like cells, as detailed below. Induction of the M cell phenotype from epithelial cells such as a clone of Caco-2 cells, the C2BBE1 cell line, seeded onto the upper face of Transwell^®^ inserts, can be performed by its co-culture with Peyer’s patch lymphocytes. Epithelial cells must be incubated for 14 days before murine Peyer’s patch lymphocytes are added to the basolateral chamber. After a further 3 days of incubation, M-cell-like function, including transport of bacteria to the basal compartment, can be observed in the transformed but not the initial C2BBE1 epithelial cells [149]. Subsequently, other studies have used C2BBE1 cells in co-culture with Raji cells to establish a more stable M cell model, a key element in the induction of intestinal mucosal immunity [150]. This classical model has been enhanced by seeding Raji cells on the inverted insert rather than freely presented on the basal compartment [148]. The inverted model allowed the lymphocyte cells to colonize the epithelial layer, adding a level of complexity to the immune response. However, further studies have suggested that the M cell conversion rate remains identical between upward and inverted models at about 8–15% [151]. To get closer to in vivo conditions, further cell models have been developed by adding the HT29-MTX to the above-mentioned system for two different purposes. The first was to study the impact of the presence of the mucus layer on toxicant mobility [152], and the second was to assess the absorption of drug molecules across the intestinal mucosa [153]. This tri-culture system sounds promising for drug development studies, especially with respect to the transport and permeation of orally administered molecules [154]. The seeding ratio of 90:10 between the Caco-2 and HT29-MTX cell lines was adopted [155] since it had been shown to be the most physiologically relevant ratio.

#### 3.2.4. Investigation of the Enteroendocrine Response of the Intestinal Barrier

This approach has an extra level of complexity for the set up since the main cell lines to be used need to be grown on separate media before being merged to answer the scientific question.

Caco-2/NCI-H716: Even though Caco-2 cells and NCI-H716 are typically grown in DMEM and RPMI medium, respectively, the two cell lines can be associated to investigate a variety of functions [156]. Cells are seeded in Transwell^®^ inserts at a ratio of 80% epithelial cells for 20% endocrine cells and cultured in DMEM for 21 days to reach full differentiation and can be kept up to day 27. The investigation of hormone secretion is realized after stimulating the apical side but not the basal side of the culture with the compound of interest and generally in low-serum RPMI medium starting on day 21. Similar conditions are used to investigate gut restriction as measured by transport of a bioactive molecule from apical to basal or basal to apical compartments.

T84/NCI-H716: NCI-H716 cells have also been associated with T84 colonic cells in other reported models [157]. Endocrine differentiation was induced after seeding NCI-H716 cells in DMEM on Transwell^®^ inserts coated with matrigel. For the co-culture systems, 90% epithelial and 10% enteroendocrine cells were mixed and seeded onto the tissue culture dish. However, this model was quite unsatisfying since the next day, when the cells were washed, it led to the loss of 80% of the poorly adherent NCI-H716 cells. An alternative co-culture was described which proposed the seeding of this cell line onto a matrigel-coated disk representing 1:49 of the dish surface area. The epithelial cell line, Caco-2 cells, had to be carefully added to the dish so that surface tension prevented the cells from entering the matrigel area. Hence, both cell types remained isolated. The co-culture of enteroendocrine cells with Caco-2 or T84 cells can be realized in DMEM or in 1:1 Ham-F12:DMEM, respectively.

Caco-2/HT29-MTX/NCI-H716/Raji-B: One quadricellular model representing the different cell types contributing to the protection of the intestinal barrier has been recently reported [158]. This model is complex since the addition of cells has to be performed in a certain order and at certain timepoints. Indeed, NCI-H716 cells are incubated for 48 h on Transwell^®^ inserts coated with matrigel to trigger differentiation. Then, Caco-2 and HT29-MTX cells have to be seeded on the apical side at a ratio of 80/10/10 (Caco-2/HT29-MTX/differentiated NCI-H716). After 14 days of culture, Raji B cells may be added to the basolateral chamber to differentiate Caco-2 into M cells. This quadricellular system may be maintained for 7 days prior to testing. The expression of MUC2, sucrase-isomaltase (SI), Matrix Metalloproteinase 15 (MMP15) and glucagon (GCG) can be assessed to confirm the presence and differentiation of the different cell types. However, little information was provided about this model for assessing its technical and functional relevance [158].

All the static in vitro models described above have provided a better understanding of intestinal epithelium development and functionalities while reducing animal testing. However, a major limitation remains in their lack of interaction with the microbiota, the study of which would require additional approaches.

### 3.3. Advanced Models of the Gut–Lumen Interactions

The interactions between the gut mucosa and luminal environment have also been extensively studied. A few researchers have investigated the combination of in vitro digestion models together with the mucosal membrane, while others have concentrated directly on bacterial interactions with the mucosa. We will discuss herein the main designs that have been used in recent years.

#### 3.3.1. Bioavailability Studies

Caco-2 cells have been associated with various models of in vitro digestion, including batch digestion [159,160], a TNO intestinal model of the upper digestive tract (TIM-1^®^) [161,162] and a simulated human intestinal microbial ecosystem (SHIME^®^) [160,163], in order to investigate the bioavailability of various molecules of interest. Caco-2 epithelial cells were cultured in DMEM medium and seeded on porous membranes in a Transwell^®^ system, except for one occurrence [159] where the cells were seeded at the bottom of 6-well plates. Filtrates from in vitro digestion and SHIME^®^ were diluted into HBSS [160,163] or DMEM [159] before treatment, while TIM-1^®^ dialysates could be tested undiluted [162]. For all the studies but one, treatment time was 4 h, and the bioactive molecules were detected in the basal compartment. However, authors do not always specify the details of the pretreatment of samples prior to exposure to cells. Often, filtrates were obtained by 0.2µm membrane filtration to remove bacteria and potential macroscopic structures before dilution 1:5 or 1:10 in the selected treatment medium. As they are still under research, standardized protocols do not seem to be available. In one study, mucin (type III, porcine) was applied to the apical side prior to treatment, in order for the epithelium to be more representative of human in vivo conditions [162]. The authors noted the impact of the mucin layer on the uptake of bioactive molecules.

#### 3.3.2. Cytotoxicity and Inflammatory Response

Cytotoxicity and inflammatory responses measured at the mucosal layer can both be linked to the bacterial environment or the bacteria themselves. A few authors have investigated the response to filtrates from upper intestine models such as TIM-1^®^ [162], but also to fecal waters or filtrates from in vitro colonic models including the TNO intestinal model of the colon (TIM-2^®^) [160,164] and SHIME^®^ [165,166]. Inflammation and barrier integrity were also measured after direct exposition to selected bacteria [167,168]. A wide range of epithelial cell lines have been described, including Caco-2 [162,167,169] and Caco-2-TC7 clone [165], and also HT29 [167], HT29-MTX [170], and CCD841 [167], as well as co-culture models such as Caco-2/HT29-MTX [168] or Caco-2/EAhy926 [160]. Bacteria were resuspended into antibiotic-free culture medium prior to treatment and added at a ratio (epithelial cell: bacteria) ranging from 1:1 to 1:1000 for periods of time ranging from 1 h (for inflammation response and barrier integrity) up to 24 h (for cytotoxicity), with a median exposure time of 3 to 4 h. Outcome measurements included tight-junction integrity, cytokine production, and expression of TLR4 proteins. However, since all these models were used in different stimulatory conditions, it remains hard to compare them.

#### 3.3.3. Adhesion, Invasion and Translocation by Probiotic and Enteropathogenic Bacteria

Bacterial colonization by fecal microbiota has been reported using HCT116 and NCM460D cell lines seeded onto wells and treated when fully confluent [65,171]. Fecal bacteria were pretreated with mucin (type III, porcine) to enrich in mucosal bacteria before suspension in antibiotic-free culture media and exposure to the cell layers for 2 h. In a similar design, Caco-2 cells were seeded onto wells and cultured until fully differentiated (14 days post-confluence) before being exposed to lactic acid bacteria for 3 h [172]. Cells were then thoroughly washed to measure the remaining adherent bacteria. Some authors investigated the ability of probiotic bacteria to prevent adhesion of pathogenic enterobacteria [170,173,174]. In all cases, colonocytes were seeded onto wells, not permeable membranes in inserts, and bacterial exposure was limited to 1 h before the epithelium was thoroughly washed to measure the remaining adherent bacteria. Different cell lines have been reported, including HT29 [173], HT29-MTX [170] and HCT15, another human colorectal carcinoma cell line [174]. Biofilms are typically visualized by crystal violet staining of gram-negative bacteria after overnight incubation with or without epithelial cells [175,176]. They are characterized by a strong interbacterial network forming a physical layer rather than isolated bacteria. Biofilm formation can be a preliminary step to select potentially pathogenic bacteria prior to mucosal exposure [174,176]. Very recently, a 3D flipwell system to grow the tri-culture was set up as an innovative tool to observe not only the effects of the molecule of interest on gut bacteria, mucus, epithelium and immune cells, but also their crosstalks. The main originality of this model was in the establishment of a multicell layer aiming at copying the intestinal mucosa [177]

Invasion and translocation experiments require the cells to be seeded onto semi-permeable membranes in cell-culture inserts (1 to 8 µm pore diameter). For invasion, authors are looking for intraepithelial bacteria. Hence, after initial exposure for the adhesion test on Caco-2 cells, the culture medium is replaced with a medium containing antibiotics for 30 min in order to kill any adherent bacteria before measurement [176]. For translocation of bacteria, the exposure time is increased to 4 and 6 h [165,178] and the bacteria are detected from the basal compartment. The test needs to be associated with a validation of membrane integrity using a permeability marker.

## 4. Further Challenges to Obtain a More Accurate Alternative Approach

From the overall review of the literature, it has been noted that the current cell models are able to partially simulate the events happening in the intestinal mucosa. The first challenge is to work on cell lines expressing the overall phenotype of their respective counterparts forming the intestinal barrier. Most of the cultured cells are partly depleted in either enzymatic activity or membrane protein expression. The second challenge is to be able to find common or compatible mediums of culture, which is, so far, the main limitation for multiple cell culture. The scientific literature describes several types of research on associating immune cells of the same species or of different species.

### 4.1. Optimizing the Co-Culture Models of the Intestinal Immune Response

Most of the cell lines and models described above associate at least one cell line of tumorigenic origin, which may impact on its response to nutrient, toxicant or drug stimulus. There is, so far, no co-culture model exclusively using human normal cell lines. There is a single study that relates to co-cultures of normal cell lines using porcine absorptive enterocyte cells, PSI, and mucin-positive enterocyte/goblet cells, CLAB, in association with macrophage cells, PoM, to create a functional intestinal system model grown in Transwell^®^ inserts, but little information about it has been published [83].

Moreover, most of the time, cell lines used in co-cultures simulating the immune response of the intestinal barrier come from the innate immune system, mainly presenting the macrophage phenotype, but less frequently of dendritic cell type and rarely of mast cell type. As specified in the previous part of the review, macrophage activation is not the only innate immune response observed at the intestinal barrier level. The investigation of macrophage/absorptive cell cooperation has to be completed by other co-culture systems involving dendritic cells or granulocytes. Among the different tools that could be used, the U937 cell line, another leukemia-derived cell line, is able to transform into macrophages after incubation with vitamin D3 and 12-O-tetradecanoyl-phorbol-13-acetate (TPA), or into dendritic cells by exposure to a self-peptide from apolipoprotein E, Ep1.B [131]. They both grow quite easily into RPMI-1640. The main advantage of the U937 cell line is that it can be cultured for more passages than THP-1. So far, to study the consequences of a chemical stimulus, THP1 and U937 are indifferently used for their ability to secrete cytokines [179,180], and to activate nuclear receptors [181] and sometimes intracellular production of ROS [182]. Another pro-monocytic human cell line, MonoMac 6, is close to mature blood monocytes thanks to CD14 antigen expression, phagocytotic ability and cytokine production when properly induced [183]. MonoMac 6 cells are used to study the inflammation-modulation activity of bioactive compounds [184]. Finally, primary dendritic cells such as BDCA3 and CD1c may also be extracted from PBMC and are easily obtained from blood. However, they have short-term survival time and the rate of extraction from PBMC is relatively low (1% of total cells), limiting their interest for the moment [185]. To overcome this limitation, the KG1 cell line could also be co-cultured. This cell line, of leukemia origin, is able to differentiate, albeit only partially, into dendritic cells [186].

Similarly, the literature is scarce, when it relates to the interaction of the gut epithelium with adaptative immune cells, due to the complexity of the physiological events leading to the activation of the T and B cells. The absence of spontaneous differentiation in many of the cell lines used is a major limiting factor, reflecting the partial inhibition of intracellular pathways. The adaptative immune response could also be investigated using transformed T-cell lines of human (HPB-ALL, HuT-78) and mouse (EL4, LBRM-33) origins. They have been used to work on T-cell receptors, but the most significant one was the Jurkat T-cell line discovered in 1980. This cell line belongs to the human acute T lymphocyte leukemia cell line. Jurkat is widely used in viral disease studies but also to evaluate the immunomodulatory potential of digestates [187,188]. From Jurkat cells, around 15 TCR-signaling mutants have been created by random mutations and selection for T-cell receptors. Jurkat cells are deficient in two lipid phosphatase expressions (PTEN and SHIP) which could alter their response to TCR stimulation [189].

Finally, the interactions existing physiologically between the immune cells, the epithelial cells and the enteroendocrine cells have not been reproduced in vitro. The lack of fully representative enteroendocrine cells models is a strong limitation for understanding them.

### 4.2. Optimizing the Technical Challenges to Strengthen the Co-Culture Models

The first factors driving the use of human cell lines are their practicability but also their easiness for extrapolating the impact on human health. It also alleviates the question of interindividual variability (although the age of the cells and the quality of the maintenance can impact the response). Models can also help decipher complex mechanisms of action that would be more difficult to isolate in vivo, or be tailored to specific scientific questions and cellular interactions. The temporality of cell-to-cell communication can also be delayed (one cell can be treated and the signaling molecules secreted into the medium can be stored until the next “organ” is ready to be treated). Finally, while the cost of cell culture is not negligible, it remains much lower than in vivo work.

The choice of cell line is associated with the scope and target of the study (a fetal cell line to observe an intestinal barrier disruption during infancy, for example). The site of investigation will also be precisely determined since intestinal and colonic barriers differ in terms of epithelial cell phenotypes, proportion of goblet cells, absence or presence of EEC, etc. To evaluate the effects of a compound on the colon epithelium, the investigators will preferably choose cell lines expressing specific markers of colonocytes, such as T84 cells [190]. Most of the epithelial cell lines used to study the intestinal barrier function come from colon cancer cells. Specific media conditions will help to orientate them to an enterocyte phenotype, such as for Caco-2. To avoid the impact of the carcinogenic pathways on the cellular mechanisms studied, and to limit the manipulation of experimental conditions, the best alternative, so far, would be to use primary fetal small intestinal epithelial cells (fSIECs) [58]. However, the investigator should keep in mind that the use of primary cells is often limited by the availability of a suitable donor and the low viability of cells in culture conditions [191].

As described above, the TEER is a common criterion to investigate intestinal homeostasis alteration. This is generally more complicated to demonstrate in vitro since the measurement is dependent upon a wide array of experimental conditions (temperature, medium formulation, cell passage, etc.). For better accuracy, internal controls for each measurement should be applied. This explains why it is generally difficult to compare experiments based on this factor alone. Thus, to confirm any change of permeability, there are other means of validating the efficacy of the tight junctions, using for example 4 kDa dextran-FITC or lucifer yellow as markers of transmembrane leakage. Future studies using the Transwell^®^ insert well plate tool will need to be methologically improved, to better reflect this intestinal barrier.

Another important challenge relates to the compatibility of cell cultures with the gut microbiota to improve the accuracy. Firstly, whenever considering microbiota to host interactions, it is important to consider the differences in culture media and the risks of unrelated bacterial contamination. As such, models will mostly involve short-term treatments where both the bacteria and epithelial cells will be exposed to a neutral environment (often HBSS buffer). Secondly, epithelial cell lines are sensitive to microorganisms which are deleterious to them. The wide diversity of microorganisms exacerbates this challenge. The sole solution today is to expose the fully differentiated cells to specific types of microorganisms compatible with the cell culture medium or with their growth medium rich in their metabolites or bacterial elements. However, this does not reproduce the physiological events since, most of the time, the co-culture happens in differentiated cell types, preventing the cells from interacting with each other from the beginning. Finally, a physiological model of colonic epithelium is required. This can be achieved by using colonic epithelial cell lines together with 20–30% goblet cells to have the full complexity of the colon crypts. Caco-2/HT29-MTX co-cultures (in a 70:30 ratio) are frequently used because of the thick mucus layer generated, even though differentiated Caco-2 cells express the machinery of small intestine not colonic epithelium. The microbiota is an integral part of the model, and the mucosa can be associated with upstream in vitro models of colonic digestion or selected bacterial strains, depending on the scientific question. A promising new in vitro static flipwell system has lately been published for investigating the interactions between gut microbiota, epithelia, and the immune system [177].

Except for in cytotoxicity studies, the rationale for a study being undertaken may necessitate using co-cultures of cells coming from different species because it allows better discrimination of the role played by each cell type on a given event at the intestinal level. In that case, the main threat would be that signaling molecules may not be suitable for intercellular communication with the different cell types and/or species.

Concerning investigations of cellular and epithelial activity, the choice of the epithelial cell line is crucial since results may differ. LS180 and Caco-2 cells are poorly suitable for assessing intestinal drug permeability and first-pass metabolism [58]. Furthermore, while some work has been done to characterize the pharmaceutical properties of both stem-cell-derived and primary human intestinal epithelial cells [192], little is known about their metabolic activity, apart from the work of the Thummel group. The authors compared the sensitivity of fSIECs, T84, Caco-2 and SL180 cell lines to drug disposition in the small intestine and concluded that none of the models fully reproduced the in vivo drug absorption and metabolism [58]. Thus, the search for an ideal in vitro model is still ongoing. The evaluation of macronutrient digestion is also an important axis of investigation and the choice of cell models may condition the quality of results. The comparison of cellular activity of different epithelial cell lines has pointed out that the differences among these cell lines is often related to differentiated expression of membrane transporters of nutrients, cell culture micro-environment or operator-bound practices [193].

Finally, the choice of different tools to realize the culture may condition the response obtained. Compatibility of cell culture medium can be an issue whenever considering co-culture models. Another technical issue for multicompartment models using Transwell^®^ inserts is the size of the pores. They need to be small (0.4 to 1 µm) for absorption studies but larger (1 to 8 µm) for the bacterial translocation of cell invasion, including re-localization of immune cells in some models. The final element to consider is the nature of the cells used, their physiological function or the cell type that they mimic when included in the various models. The different parts of the digestive tract (small intestine or colon, for example) do not have the same composition, and having a cell type overrepresented in the model could be detrimental to the relevance of the system. Researchers should also not forget the adjustment of differentiation and functionalization timelines between the different cell types that will condition the results obtained.

## 5. Conclusions

Reproducing interactions of the different cells present in the gut to simulate the intestinal barrier function remains a major challenge to be able to mimic and understand the impact of the consumption of nutrients, toxicants or drugs on gut homeostasis and the pathophysiological cellular and molecular pathways that may be related to these substances.

As we discussed in this review, approaches using cell lines have been common for many years and are mostly satisfactory. Originally, these approaches were based on the use of single-cell types, essentially enterocytes, and these can be useful for studying some functionalities such as absorption. However, the need for biological answers closer to the in vivo conditions required some complexification of the models. We saw that the addition of mucus cells, but also of immune cells or of bacteria with absorptive cells, has gained popularity, but this can lead to many technical issues, among which are culture conditions, particularly when growing prokaryotic with eukaryotic cells.

Investigations are still needed to reproduce in vitro a maximum of the in vivo functionalities of each cell type in culture, but also of the interactions existing in a whole organism. This will be achieved by testing the specific response of the modelled system and by using technical solutions such as the Transwell^®^ insert or matrigel, especially when targeting reproduction of the polarization of the gut. While not mentioned in this review, 3D gut organoids have been used in recent years. Developed from adult pluripotent stem cells to form spheroid structures, they retain intestinal regional identity (of the small intestine or colon) but contain only epithelial cells. Furthermore, the lumen is inside the structure; the compounds of interest thus need to be microinjected. All these constraints limit their interest for understanding complex mucosal interactions [194]. In addition, their inward polarization makes most organoid systems hard to challenge without microinjection procedures to mimic apical to lumen interactions. Organoid disruption into a 2D insert format is easier to test but seems like a step back. Apical-out designs are slowly emerging [195,196] to study host–pathogen interactions. In some cases, particularly for extra-intestinal interactions such as brain–gut axis, we cannot be sure that it will ever be possible to model them accurately using only static systems. The use of dynamic systems combining a model of intestinal mucosa exposed to other cell types, whether including or not signaling molecules, will allow the creation, at least partially, of a more representative model. Lastly, organ-on-a-chip (OoC) technology gives the opportunity for multiple organ communication, with fluidic channels creating a dynamic and interactive environment for the static 3D organ chambers. However, while there are numerous examples of OoCs being developed around the world, the complexification is not a simple matter of connecting existing systems [191]. The advanced understanding of complex cellular interactions remains of major interest and is a steppingstone for deepening our knowledge of gut homeostasis mechanisms. It can also be of interest for generating more data for in silico molecular and cellular simulation using artificial intelligence to model the complex networks.

## Figures and Tables

**Table 1:** Main epithelial cell lines.

	Cell Line	Origins	Culture Conditions	Use	Specificities	Reference
Enterocytes	Caco-2 (HBT-37)	Human colorectal adenocarcinoma	RPMI/DMEM + 10% FBS maintenance 1:10 dilution upon reaching 80% confluence culture on inserts: 4 × 10^5^ cells/cm^2^ spontaneous differentiation when reaching confluence (14–21 d)	Permeability, absorption, transepithelial transport after oral intake	Apical brush border with microvilli and tight junctions brush border activity: lactase, aminopeptidase N, sucrase isomaltase, dipeptidylpeptidase IV No mucus secretion Molecular transport: amino acids, sugars (GLUT1, GLUT2, GLUT3, GLUT5, SGLT1), vitamins (B12, D3), hormones, xenobiotics (P-gp, MRP), but low PepT1 Drug metabolization: aminopeptidase, esterase, sulfatase, glucuronosyltransferase, CYP1A1 or CYP1B1 but not CYP3A4 or CYP2B6 Inflammation: IL6, IL8, TNFa, TGFb1, TLSP, IL15, low TLR-4	[27,28,29,30,31,32,33]
	Caco-2/TC7	Clone of Caco-2 from late passage (198)	DMEM 10% FBSDMEM + HEPES + 10% FBS low glucose consumer, differentiation faster than parental line (20 d vs. 30 d), homogeneous population	Intestinal drug permeability and absorption studies	Well-organized brush border and high sucrase isomaltase, DPP-IV TLR2 + TLR4 Molecular transport: SGLT1, GLUT2, GLUT5 remain high but GLUT1 and GLUT3 decrease after confluence, good P-gp Drug metabolization: increased DMT1, glucuronosyltransferases, low transferrin receptors, CYP1A1 but no CYP3A4 (except MTX-induced)	[27,34,35,36,37,38,39]
	Caco-2/C2BBE1	Clone of Caco-2		Glutamin-induced carcinogenesis	Good level of sucrase isomaltase, well-developed brush border, all microvillar proteins expressed including myosin I Molecular transport: folate receptor, vit C channel	[27]
	Caco-2/15	Clone of Caco-2 vit D3-sensititve			Inhibition of cell growth in response to vit D3 High sucrase isomaltase activity	[27]
	HT29	Human colon adenocarcinoma	DMEM + 10% FBS McCoy’s 5A + 10% FBS; differentiation in glucose-free medium very similar to Caco-2 after 30 d	Cell differentiation, food digestion and bioavailability Drugs and food transport Intestinal immune response to bacterial infection, microorganism survival, adhesion or invasion	Considered a pluripotent intestinal cell line Brush border: aminopeptidase N, dipeptidyl peptidase-IV and alkaline phosphatase and villin, low sucrase isomaltase, no lactase MUC1 expression except at late confluency Cytokines: TNFα, IL1β, IL6, IL8, IL15, VEGF, GCF, GMCF, IL3 No PYY and NPY receptors MCT1 transport Impaired glucose metabolism; brush-border-associated hydrolases	[30,37,38,39,40,41,42]
	HT29-18C	Clone of HT29-	DMEM + 10% FBS + HEPES	Linear model for paracellular transport route close to ileum	Slow acquisition of electrical resistance over 24 days on inserts, polarization but poorly developped microvillus membrane	[43,44]
	HIEC-6	Human fetal cell line small intestine, normal	OptiMEM + HEPES + 4% FBS 8–10 d for differentiation	Physiological and pathophysiological conditions and interaction between intestinal epithelium and mycotoxins	Non-malignant Non-tumorigenic mimics in vivo condition better than Caco-2 PepT1 transporter Lipid-binding transport proteins iFABP	[29,45,46,47]
	tsFHIFHs-74-Int	Human fetal small intestine epithelial cells (transformed with SV40)	OptiMEM + HEPES + 4% FBS DMEM + HEPES + 7% FBS		Non-malignant At 37 °C, the SV40 antigen breaks down, leading to growth arrest and acquisition of an enterocyte-like phenotype, although they remain morphologically immature and do not form tight junctions or polarize	[48,49,50,51]
	H4	Human fetal primary intestine cell line	DMEM + 10% FBS DMEM + HEPES + 10% FBS + insulin	Inflammation response Physiopathology of necrotized enterocolitis Fatty acids transport and absorption studies	Non-malignant Highly tolerant Express more IL-8 than Caco-2 with and without LPS Not forming tight junctions or polarize	[48,52,53,54]
	CCD 841 Con = CRL1790	Normal fetal human colon tissue	DMEM/MEM + 10% FBS MEM + 3% FBS	Inflammatory mechanisms in colonic epithelial cells	Non-malignant, no keratin Stretched epithelial-like cells growing as a flattened and disorganized layer in culture Secretion TNFα, IL1β, IL8 in response to bacterial treatment	[55,56]
Colonocytes	SK-CO15	Colorectal adenocarcinoma	DMEM + Ham’s F-12 + 10% FBS	Model for Na(+)/h(+) exchange Cell migration Epithelial cyst formation Epithelial barrier function Transport studies Regulation of epithelial junctions	Expression of NHE1, 2, 8 and 3 and of NHERF1 and NHERF2 Polarized distribution similar to original glycoprotein antigens in human colon High expression of NHE3 No expression of sucrase-isomaltase, aminopeptidase N, lactase, glucoamylase Absence of brush border, low alkaline-phosphatase and villin levels Few and disorganised microvilli	[57]
	T84	Lung metastasis colon carcinoma	DMEM/Ham’s F-12 + 10% FBS or 5% NBS	Hormonal control of colon cell growth Electrolytes transport Intestinal permeability Inflammatory response Bacteria/enterocyte interaction	Shorter microvilli Tight junction structure, basal CYP3A4 expression controversial but PXP-induced PgP efflux No IL8 secretion in response to LPS but ok after IL1β	[30,39,58,59]
	SW620	Dukes C colorectal cancer patient, Stage 3	DMEM/10%FBS, McCoy’s 5A + 10% FBS	Drug toxicity, cancer research	Keratin IL8 secretion in response to LPS and IL1β Lower expression of MUC2, MUC6 and MUC5B with high expression of MUC1	[60,61,62,63]
	Colo-205	Ascitic fluid derived from colon cancer Dukes’ type D	RPMI-1640 + 10% FBS	Cancer research and toxicology	Keratin It has mixed growth properties: adherent and suspension	[62,64]
	HCT116	Colon cancer Stage 1 (primary)	RPMI + 10% FBS DMEM + 10% FBS McCoy’s 5A + 10% FBS	Cancer research and toxicology	Expression of apoptosis genes: Grp78, Bcl-2, NF-kappaB(p50), NF-kappaB(p65), thioredoxin peroxidase (peroxiredoxin) 2, peroxiredoxin 4, maspin, guanylate cyclase activating protein-1, PKCzeta, EGFR, Ras family members, PKA, PI(4,5)K, TRAF2 and BIRC1 (IAP protein) Mutation in codon 13 of the ras-proto-oncogene	[60,64,65,66]
	HRT18	Large intestine adenocarcinoma	RPMI-+ 10% horse serum	Cancer research and toxicology	Identical to HCT-8 cell line Epitheloid morphotype	[67]
	FHC (CRL1831)	Human fetal colon cells	Ham’s F12 + DMEM, + HEPES +10% FBS, + cholera toxin + insulin, + transferrin + hydrocortisone			[68]
	NCM460	Human normal colonic epithelial cell line	M3 medium + 10% FBS	Intestinal barrier Model for transverse colonic crypts	Immortalized Proteasome activity; characteristics of native colonocytes Gastrin-releasing peptide receptor	[69,70,71,72,73]
	CCD-18co = CRL-1459	Infant colon non-cancerous	DMEM/EMEM + 10% FBS	Cancer research and toxicology	Fibroblast morphology Senesce at about 42 PDL	[74]

**Table 2:** Main secreting cell lines.

	Cell Lines	Origins	Culture Conditions	Use	Specificities	Reference
Goblet cells	HT29-MTX	HT29 + stepwise adaptation with increased concentration of Methotrexate	DMEM + 10% FBS	Mucus-inducing properties of food compounds, microorganism adhesion survival, transport studies	MTX-induced differentiation into entirely differentiated goblet-cell-like phenotype Mucin: MUC1 and MUC5 but low MUC2 Passive diffusion CYP3A4 in apical localization	[35,39,85,86,87,88,89]
	LS180	Colon adenocarcinoma stage 2 (primary)	DMEM/Ham’s F12 + 10% FBS MEM 10% FBS	Mucin synthesis studies	Mucus-secreting cell line Expression of mucin Basal and inducible CYP3A4 activity but poor tight junction structure	[90]
	LS174T	Trypsined variant from LS180	DMEM + 10% FBS	Chemical, physiological, pharmacologic and immunologic characteristics of neoplastic colonic cells	Abundant microvilli Intracytoplasmic mucin vacuoles High level of carcinoembryonic antigens High expression of MUC2, MUC6 and MUC5B with lower expression of MUC13	[63]
Enteroendocrine cell lines	NCI-H716	Lymphoblast morphology isolated from colorectal adenocarcinoma	RPMI + 10% FBS-Need of Matrigel for adherence	Human intestinal L cell GLP-1 secretion assay in response to nutrients	Muscarinic receptors PYY and GLP-1 secretion	[91,92,93,94]
	HuTu-80	Duodenum cancerous	DMEM/F-12 + 10% FBS	Cancer research	The cells express receptors for bombesin at up to 6000 sites per cell PYY expression is reduced for butyrate or propionate stimulation	[95]
	STC-1	Murine duodenal enteroendocrine tumor L-cell Duodenal proinsulin-polyoma (transformed SV40)	DMEM + 17.5% FBS	Representative of L-cell Used to study GLP-1 secretion	Enteroendocrine cell model Express and secrete CCK, PYY, pancreatic polypeptide, neurotensin, GLP-1, GLP-2, oxyntomodulin Poor secretion of GIP	[96,97]
	GLUTtag	Murine colon tumor expressing SV40 large T antigen under control of proglucagon promoter (transformed SV40)	DMEM + 10% FBS	GLP-1 secretion	GLUT and SGLT1 glucose transporters to stimulate GLUT secretion GPR40, GPR120, TGR5, GPR43	[98,99,100]

**Table 3:** Main immune cell lines.

Cell Line	Origins	Culture Conditions	Use	Specificities	References
THP-1	Human monocytic leukemia	RPMI1640 + 10% FBS	Immune modulation and inflammation studies	PMA-induced differentiation into an adherent macrophage phenotype and further polarized into M1 and M2 macrophage cells; culture in serum-free medium + IL4, GM-CSF, TNFα and ionomycim to differentiate into dendritic-cell-expressing CD83, CD80, CD86, CD40, CD206, CD209, CD120a, CD120b + intracellular IL10	[131]
RAW264.7	Murine tumour induced by the Abelson leukaemia virus	RPMI/DMEM + FBS	Oxidative stress	Closely mimic bone-marrow-derived macrophages in terms of cell surface receptors and response to microbial ligands via TLRs IL12 NO	[129]
Raji	B-cell Human Burkitt’s Lymphoma	DMEM without Pyruvate + 10% FBS + plasmocin	Useful model for drug delivery studies	In co-culture, transform Caco-2 to reach morphological and functional features of M cell Fc receptors for binding immunoglobulin IL-10 TNF-α INF-γ lack of differentiation	[39,132]
HL-60	Promyeoloblasts from peripheral blood with promyelocytic leukemia	RPMI-1640 + 10% FBS/DMSO 1,25% for neutrophilic differentiation	Immune disorder and immunology research	Pro-monocytic cell line differentiating into macrophage or dendritic cells in the presence of butyrate, hypoxanthine, phorbol myristic acid, DMSO, actinomycin D and retinoic acid stimulation; phagocytic activity, Fc expression	[133,134]
U937	Human leukemia from histiocytic lymphoma	Differentiation into macrophages and mature monocytes with PMA-treatment (48h). Differentiating into DC-like cells by exposure to Ep1, B	Study of anti-inflammatory properties of food bioactive, monocyte–endothelium mechanism of attachment, monocyte–macrophage differentiation	Pro-monocytic cell line that can differentiate into macrophage or dendritic cells with more passage stability than THP-1	[131]

## Data Availability

Data sharing not applicable.
